# Reconstruction of a secondary scalp defect using the crane principle and a split-thickness skin graft

**DOI:** 10.1186/s12893-021-01056-y

**Published:** 2021-01-18

**Authors:** Yi Lu, Ke-Chung Chang, Che-Ning Chang, Dun-Hao Chang

**Affiliations:** 1 School of Medicine, National Yang-Ming University, Taipei, Taiwan; 2 Division of Plastic and Reconstructive Surgery, Department of Surgery, Far Eastern Memorial Hospital, No. 21, Sec. 2, Nanya S. Rd., Banciao Dist., New Taipei City 220, Taiwan, ROC; 3 Division of Plastic and Reconstructive Surgery, Department of Surgery, Taipei Veterans General Hospital, Taipei, Taiwan; 4 Department of Information Management, Yuan Ze University, Taoyuan City, Taiwan

**Keywords:** Crane principle, Scalp reconstruction, Local scalp flap

## Abstract

**Background:**

Scalp reconstruction is a common challenge for surgeons, and there are many different treatment choices. The “crane principle” is a technique that temporarily transfers a scalp flap to the defect to deposit subcutaneous tissue. The flap is then returned to its original location, leaving behind a layer of soft tissue that is used to nourish a skin graft. Decades ago, it was commonly used for forehead scalp defects, but this useful technique has been seldom reported on in recent years due to the improvement of microsurgical techniques. Previous reports mainly used the crane principle for the primary defects, and here we present a case with its coincidental application to deal with a complication of a secondary defect.

**Case report:**

We present a case of a 75-year-old female patient with a temporoparietal scalp squamous cell carcinoma (SCC). After tumor excision, the primary defect was reconstructed using a transposition flap and the donor site was covered by a split-thickness skin graft (STSG). Postoperatively, the occipital skin graft was partially lost resulting in skull bone exposure. For this secondary defect, we applied the crane principle to the previously rotated flap as a salvage procedure and skin grafting to the original tumor location covered by a viable galea fascia in 1.5 months. Both the flap and skin graft healed uneventfully.

**Conclusions:**

Currently, the crane principle is a little-used technique because of the familiarity of microsurgery. Nevertheless, the concept is still useful in selected cases, especially for the management of previous flap complications.

## Background

Scalp defects result from several etiologies, such as trauma, infection, neoplasm ablation or congenital deformities. Since scalp defects may be partial or full-thickness, different surgical methodologies and reconstruction approaches are considered that involved multiple dimensions, such as the type of defect, patient characteristics, and surgeon preference [1]. In 1955, Figi and Struthers were the first to report on the temporary use of a scalp flap placed over an exposed skull defect to provide immediate blood supply and coverage, and the eventual deposition of a layer of soft tissue, which is later used to nourish a skin graft and the scalp flap is later returned to its original location. In 1969, this method was named the "crane principle" by Millard [2]. As for scalp reconstruction, the crane principle takes advantage of the five-layer structure of the scalp and is a relatively simple procedure to utilize [3], especially in the era before microsurgery.

Here we present a case of a 75-year-old female patient with a temporoparietal scalp squamous cell carcinoma (SCC). After wide excision of the skin cancer along with the underlying pericranium, the primary defect with skull exposure was reconstructed by transposition flap and split-thickness skin graft (STSG) on the pericranium of the flap donor site. Unfortunately, the patient experienced a postoperative complication with skin graft loss and bone exposure. For this secondary defect, we utilized the crane principle to rotate the previously placed flap and leave a layer of soft tissue as a salvage procedure and skin grafting to the original tumor location. Ultimately the flap and skin graft healed well and they both were in stable condition in the following 6 months. This case report was approved by the Research Ethics Review Committee of Far Eastern Memorial Hospital (FEMH, New Taipei City).

## Case report

**Fig. 1:**
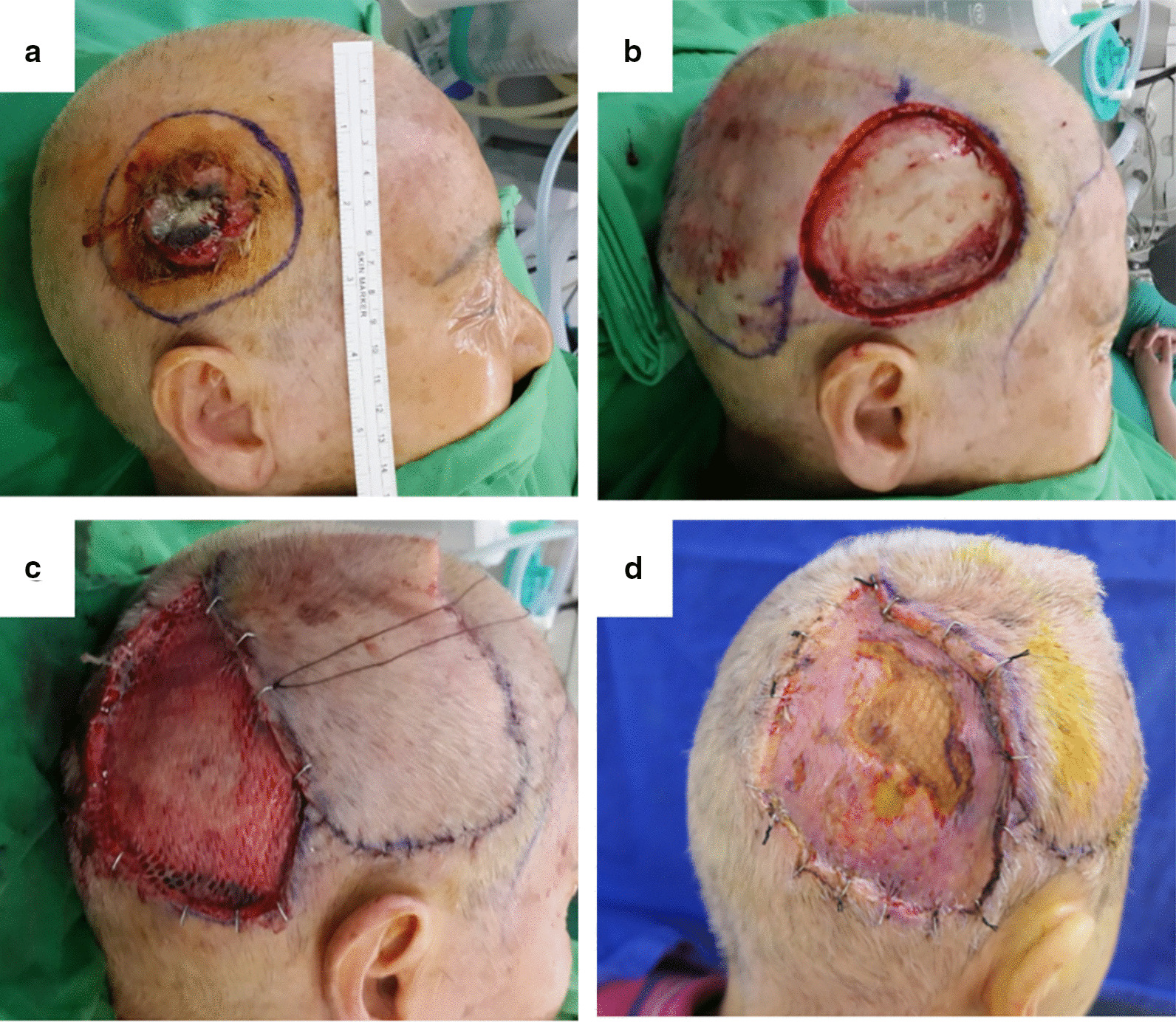
**a** A 75-year-old woman with a 4 × 4 cm squamous cell carcinoma on the right temporoparietal scalp. **b** Wide excision with 1.5 cm margin was performed and deep to the scalp bone. **c** The defect was reconstructed with transposition flap, and the donor site was covered with meshed STSG. **d** Partial skin graft loss was noted after tie over bolster removal

A 75-year-old woman presented a 4 cm × 4 cm protruding ulcerative skin lesion on the right temporoparietal scalp (Fig. 1a). She had a history of lobectomy for lung adenocarcinoma 10 years ago and was in stable condition. After a biopsy, the lesion was proven to be SCC. Physical examination and computed tomography (CT) scan showed no lymphadenopathy in the preauricular, postauricular, or cervical regions, but the tumor invaded deeply, which just abutted the pericranium. Wide excision with a 1.5 cm margin was performed and the dissection plane was deep and directly under the pericranium layer, which left a 7 cm × 7 cm defect with bare bone exposure (Fig. 1b). The defect was reconstructed using a cephalically-based transposition flap that was harvested from the occipital area. The flap donor site, with an intact pericranium, was resurfaced with meshed STSG and fixed with a tie over bolster dressing (Fig. 1c). The final pathology report revealed moderately differentiated, pT3 SCC without lymphovascular or perineural invasion, and both the peripheral and deep margins were free from tumor invasion.

During the follow-up period, a partial loss of the STSG occurred on the center of flap donor site. After conservative wound care for 45 days, the wound was revealed to be a skin and soft tissue defect with bare bone exposure (Fig. 1d). After discussions with the patient and her family, a decision was made to rotate the previous flap back with the galea and a layer a soft tissue left in situ and then the skin graft was performed on the original tumor location, so called as the “crane principle”.

**Fig. 2:**
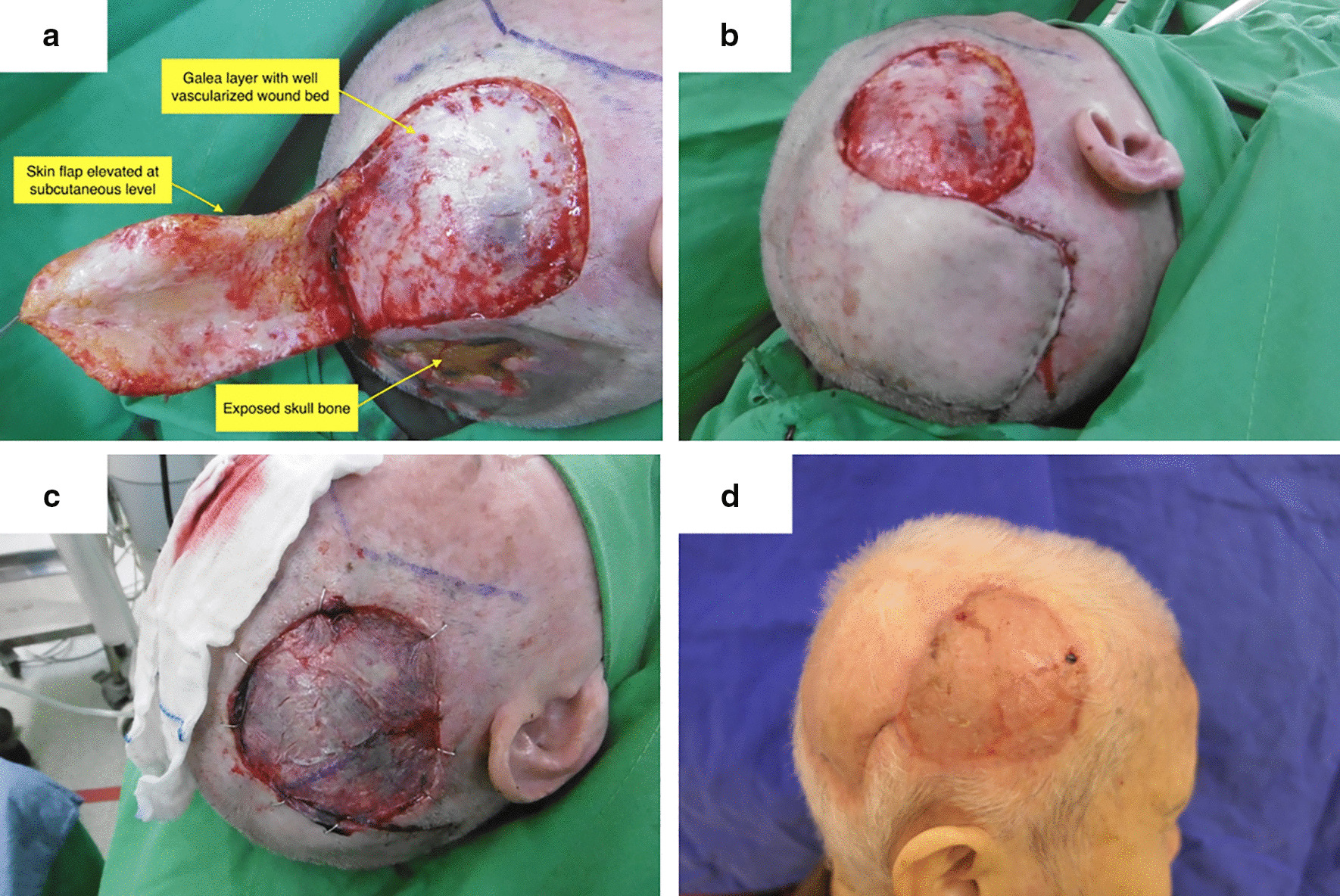
**a** Bone exposure at occipital wound was noted at 1.5 months post tumor resection. **b** The flap was rotated back to cover the occipital bone exposed wound. A thick layer of well-vascularized soft tissue was noted at the temporal wound. **c** The wound was covered by STSG harvested from the adjacent scalp. **d** Both flap and temporal skin graft healed well at the 2-week follow-up

During the surgery, we injected 1:200,000 epinephrine into the subcutaneous layer and the flap was elevated with a sharp dissection between the subcutis and galea. (Fig. 2a) After debridement of the occipital wound, the flap was rotated to cover the wound. (Fig. 2b) A thick layer of well-vascularized soft tissue was left in the temporoparietal region, and the wound was covered by STSG harvested from the adjacent scalp (Fig. 2c).

The flap and skin graft healed well at postoperative 2 weeks. (Fig. 2d) There was no tumor recurrence at both the 3-month and 6-month follow-up.

## Discussion

SCC is a malignant and invasive neoplasm, which can potentially present with distant metastases. Overall, 3–8% of SCCs are located on the scalp [4]. In this particular location, SCC is clinically characterized with a greater tendency toward ulceration. Studies report that there is a relatively higher probability of chronic, non-healing occurrence of ulcers compared to other skin locations [5]. Hence, radical excision followed by reconstruction has become the standard treatment to scalp SCC [6]. In our patient, the preoperative CT scan showed deep invasion of the tumor without skull bone involvement. Therefore, based on the “non-touch” policy, we excised the tumor subperiosteally. The final pathological findings also confirmed the adequacy of the excision margins.

There are many surgical techniques for scalp reconstruction, including primary closure, skin grafting, local flaps, regional flaps, free tissue transfer, and tissue expansion [7]. Several factors should be taken into consideration regarding the selection of the technique, such as defect thickness, size, location, the status of pericranium and calvarial defects, prior surgical procedures and the medical and functional status of the patient [8].

In our case, the patient was of older age and had restricted lung function due to the previous lobectomy. Given these factors, after well discussion and consent, we chose to use a transposition flap with skin grafting for her scalp reconstruction rather than other more complicated and time-consuming techniques. The reason why we selected the occipital scalp as the donor site was to hide the alopecia area on the rear of her head. However, the donor site wound ended up with subsequent graft loss, probably related to compression during rest or sleep resulting in loss of blood flow to the STSG.

**Fig. 3:**
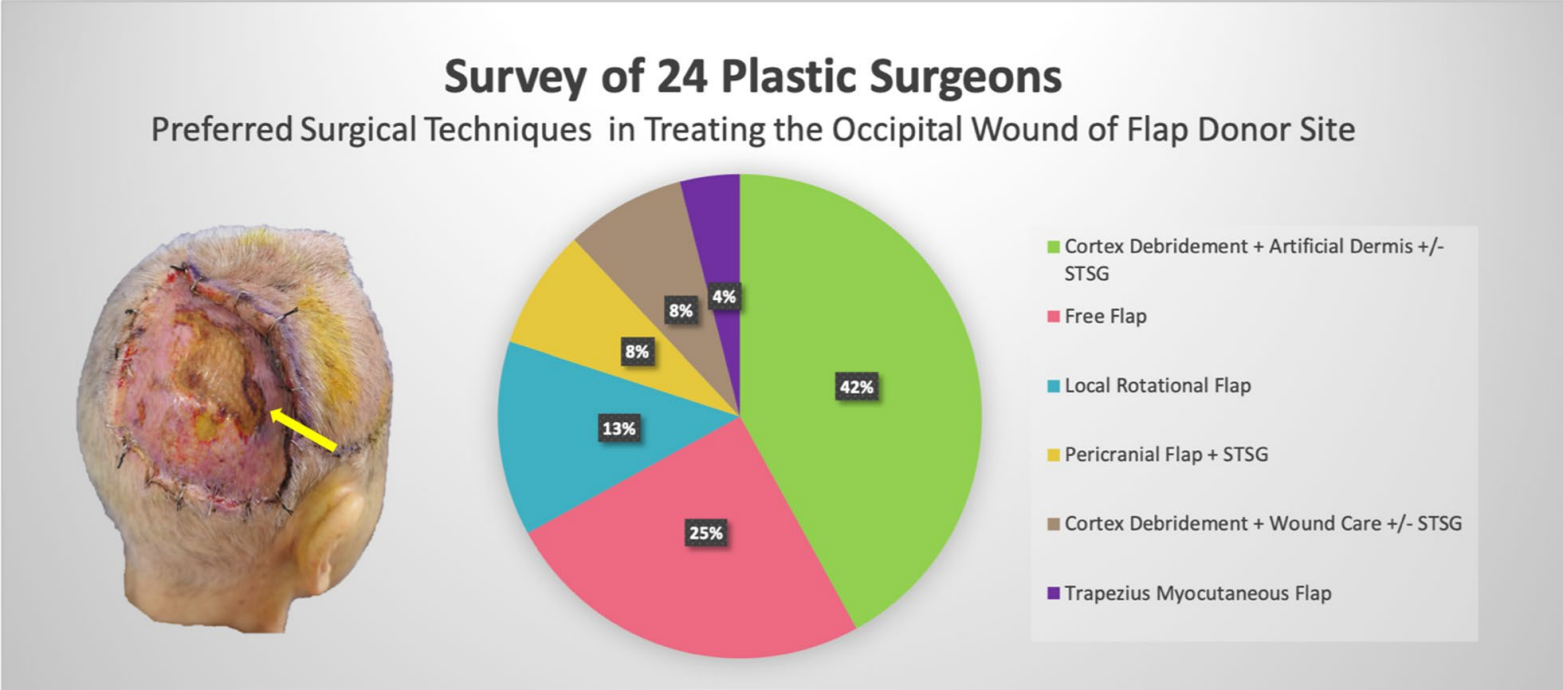
The preferred surgical techniques for the complication management (occipital wound after flap transfer) in the survey of 24 plastic surgeons

In this report, our focus is not on the primary scalp reconstruction but rather on the treatment strategy for the complication that developed after the first reconstruction. For this bone exposing occipital wound, there were some other possible solutions [9–11]. We also conducted a survey according to this specific scenario among the board-certificated plastic surgeons in our society. Twenty-four plastic surgeons completed the survey, and their preferred (first-considered) surgical techniques were shown in Fig. 3. Most of the surgeons preferred more conservative methods, such as removing or drilling the outer cortex + artificial dermis (45.8%) or wound care (8.3%) ± later STSG. However, some considered that these techniques do not guarantee stable graft take and subsequent wound healing and therefore would choose flap reconstruction, including free flaps (25%), local rotational flap (13%), trapezius flap (4%) and pericranial flap + STSG (8%). Not surprisingly, no one mentioned the crane principle. Nevertheless, after we introduced it, 62.5% of the surgeons would consider this technique as a priority choice. We believed that the crane principle was the easiest, most straightforward, and promising method to deal with this complication. The returned flap covering the occipital area could also better tolerate the pressure of lying down, and a skin graft on the temporal area is much easier to care for.

The original goal of the crane principle proposed by Millard in 1969 was to shorten the interval of pedicle division of the abdominal flap in hand reconstruction [2]. The abdominal flap was transferred back seven days later, leaving a thin fascia and granulation tissue on the exposed tendon or bone of the hand for subsequent STSG [2]. This method also avoids the need for multiple defatting procedures in the standard abdominal flap. Ship et al. and Wolfe extended the crane principle from hand surgery to scalp reconstruction [12, 13]. The most common scenario for using the crane principle in their reports was to use hair-bearing scalp to reconstruct a forehead full-thickness defect. The interval between the flap transfer was about one month and 3–4 months total when combined with bone graft reconstruction. Recently in 2020, two reports about crane principle have been published. Dhar et al. in Bengal reported a case of scalp degloving injury treated with the crane principle over an 8-month interval [14]. Kadry et al. in Egypt presented a case series of twenty patients with scalp trauma or electric burn injuries [15]. Their interval between flap transferals was only two weeks. They used STSG to cover the donor site at the first stage, and the skin graft was later taken down at the second stage to cover the original defect area which was carpeted with a well-vascularized galea layer. In their series, three patients had wound dehiscence and two had flap donor site grafts ulceration. In our case, we didn’t re-use the skin graft on the original donor site, because the graft was tattered and fragile. On the contrary, we harvested STSG from adjacent scalp to achieve better wound healing.

What mechanism makes the crane principle work? The pathophysiology has not been clearly described in previous reports. In Millard’s experiment of dogs, he used a trapdoor flap to cover a periosteum-removed rib [2]. After one week, the subcutaneous patch was adhered to the edges of surrounding tissue but could be lifted from the underneath rib, indicating the edge to edge circulation build-up. Another concept had been proposed by Mitnoun in 1989, called the “nutrient flap” [16]. He transferred free flaps to the patients with lower limb ischemia, providing the supplementary blood flow to the distal ischemic zone. The angiography at 3 weeks revealed neovascularization of the capillary bed over the previously ischemic defects. In the scalp cases, such as our patient, usually had larger bone exposed area than Millard’s rib model. If only waiting for one week, it may not be enough to supply the whole area by edge to edge circulation. Therefore, based on the nutrient flap concept, it’s reasonable to take longer time interval between flap transfer in scalp crane technique, at least 2 weeks or longer. This was also proven in our case and Ship’s, Wolfe’s and Kadry’s studies.

In the last 30 years, due to the popularization and progress in microsurgical techniques, the crane principle has seldom been reported in the literature. Most plastic surgeons nowadays are not familiar with this principle. Nevertheless, the concept is still useful in selected cases, especially in the management of the complication of the previous flap [17].

In conclusion, our original intention was not to use this older technique to complete the scalp reconstruction, but it was a viable solution to the loss of the skin graft complication. In medicine, sometimes the adage “the older the wiser” is true.

## Data Availability

All patient data and clinical images adopted are contained in the medical files of Far Eastern Memorial Hospital. The data supporting the conclusions of this article are included within the article and its figures and tables.

## Abbreviations

SCC: Squamous cell carcinoma
STSG: Split-thickness skin graft
CT: Computed tomography
